# Species-Specific Differences in Aryl Hydrocarbon Receptor Responses: How and Why?

**DOI:** 10.3390/ijms222413293

**Published:** 2021-12-10

**Authors:** Xiaoting Xu, Xi Zhang, Yuzhu Yuan, Yongrui Zhao, Hamza M. Fares, Mengjiao Yang, Qing Wen, Reham Taha, Lixin Sun

**Affiliations:** 1 Jiangsu Center for Pharmacodynamics Research and Evaluation, China Pharmaceutical University, Nanjing 210009, China; 3219071126@stu.cpu.edu.cn (X.X.); 3219071117@stu.cpu.edu.cn (X.Z.); 3320071816@stu.cpu.edu.cn (Y.Y.); 3320071853@stu.cpu.edu.cn (Y.Z.); 3719019003@stu.cpu.edu.cn (H.M.F.); 3220070785@stu.cpu.edu.cn (M.Y.); 3220070801@stu.cpu.edu.cn (Q.W.); 2020182802@stu.cpu.edu.cn (R.T.); 2 Key Laboratory of Drug Quality Control and Pharmacovigilance, China Pharmaceutical University, Ministry of Education, Nanjing 210009, China

**Keywords:** aryl hydrocarbon receptor, species differences, mechanism, ligand-binding domain, dioxin response element

## Abstract

The aryl hydrocarbon receptor (AhR) is a transcription factor that regulates a wide range of biological and toxicological effects by binding to specific ligands. AhR ligands exist in various internal and external ecological systems, such as in a wide variety of hydrophobic environmental contaminants and naturally occurring chemicals. Most of these ligands have shown differential responses among different species. Understanding the differences and their mechanisms helps in designing better experimental animal models, improves our understanding of the environmental toxicants related to AhR, and helps to screen and develop new drugs. This review systematically discusses the species differences in AhR activation effects and their modes of action. We focus on the species differences following AhR activation from two aspects: (1) the molecular configuration and activation of AhR and (2) the contrast of cis-acting elements corresponding to AhR. The variations in the responses seen in humans and other species following the activation of the AhR signaling pathway can be attributed to both factors.

## 1. Introduction

The cytoplasmic receptor aryl hydrocarbon receptor (AhR) is a member of the primary region helix–loop–helix Per-Arnt-Sim (bHLH/PAS) family, which can bind with some exogenous (polyhalogenated aromatic hydrocarbons, polychlorinated biphenyls, and polycyclic aromatic hydrocarbons) and endogenous (indirubin, 6-formylindolo [3,2-b] carbazole, kynurenic acid, and indole) chemicals in the cytoplasm [1,2] After activation, AhR relocates from the cytoplasm to the nucleus and initiates the expression of several genes, producing dissimilar biophysiological and toxicological effects in the tissues of a wide range of mammalian and nonmammalian species [3,4,5,6]. In the AhR protein amino acid sequence, the N-terminus is extremely conserved among species [7], and the modes of action of AhR are also similar. However, several observations have claimed that the same AhR ligand can exert unique toxicological and physiological responses in different species [8,9]. Early studies focused on the toxic response to xenobiotics mediated by activated AhR. However, recent studies have illustrated the endogenous regulatory role of AhR in normal physiology and development, such as the modulation of immunity, hematopoiesis, normal skin formation, intestinal barrier, and neurogenesis [10,11,12,13,14]. These versatile roles have highlighted AhR as a promising therapeutic target. Although recent studies have shed much light on AhR from various perspectives, the physiological functions of AhR in humans are still unclear. The main obstacle to understanding AhR function lies in its high cell and species specificity. Understanding these differences and mechanisms of the AhR response can help us select more appropriate animal models and provide a new understanding that helps in rational targeted AhR drug design. The hypothesized physiological functions of AhR in humans can be defined and strengthened by comparing the functions of AhR among species.

## 2. Structure and Mechanism of AhR Action

**Figure 1:**
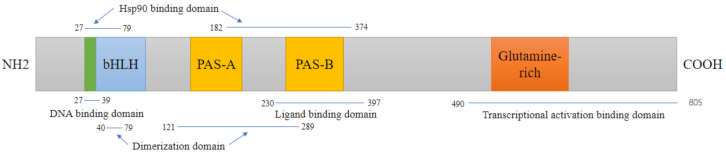
Domain structures of mouse AhR. The AhR protein contains several critical domains. DNA binding domain:The basic region helix–loop–helix (bHLH) aids in binding the transcription factor to DNA and protein–protein interactions; Ligand binding domain: PAS B domain along with flanking amino acid residues on both sides, serves as ligand-binding domains, and mediates interactions with several other proteins;. Transcriptional activation binding domain: In the C-terminal region, a glutamine-rich domain is involved in coactivator recruitment and transactivation.

**Figure 2:**
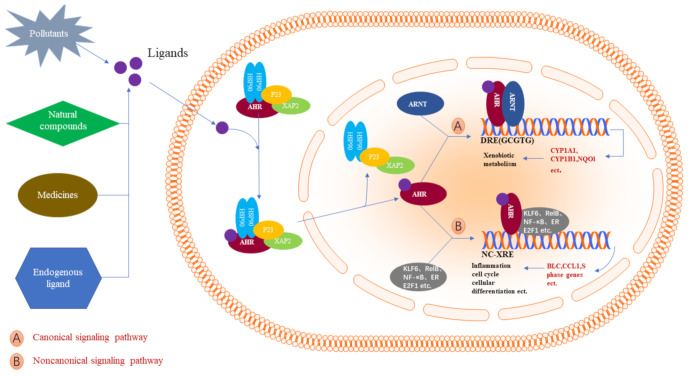
AhR activation mechanisms in mammalian cells. The inactive form of AhR is retained as a complex with chaperone proteins, including HSP90, XAP2, and P23, in the cytoplasm. Many exogenous AhR ligands, such as environmental pollutants, natural compounds, various drugs, and some endogenous substances, induce a conformational alteration in AhR to activate nuclear transport. In the nucleus, AhR works through two signaling pathways. In the canonical pathway, AhR and ARNT binding expose 5′-GCGTG-3′ as the core consensus motif. Subsequently, it regulates its target genes, such as *CYP1A1, CYP1A2,* and *NQO1*, which are involved in xenobiotic metabolism. Noncanonical signaling occurs mainly through interactions with other regulatory proteins, such as KLF6, RelB, and NF-κB, which are involved in cell cycle regulation, growth differentiation, and the inflammatory response.

The helix–loop–helix (bHLH) domain in AhR is the most functional domain. It is located in the amino(N)-terminal half of the AhR and contains a DNA-binding region that can specifically recognize and interact with the dioxin response element (DRE) sequence of the target gene. bHLH is significantly conserved between species, and the amino acid sequence similarity between rats/mice and rodents/humans reaches 100% and 98% [15,16], respectively. Following two Per-Arnt-Sim (PAS) domains (A and B), each of them plays different roles: formation of the heterodimer with the AhR nuclear translocator (ARNT) is due to the involvement of the PAS A domain, while the PAS B domain, along with flanking amino acid residues on both sides, is closely related to the binding of ligands, which are collectively called the AhR ligand-binding domain (AhR-LBD). The AhR-LBD amino acid sequence is 97% conserved between mice and rats, and both species share 85–87% identity with humans [17]. However, the minor difference in the amino acid sequence in the AhR-LBD structure dramatically influences AhR ligand binding. On the other hand, the C-terminal region of the AhR transactivation domain (TAD) shows only 58% similarity between humans and mice [18]. TAD comprises smaller subunits, proline/serine/threonine-rich (P–S–T-rich) subregions, acidic subregions, and glutamine-rich (Q-rich) subregions. The Q-rich subregion appears to have the greatest transactivation potency and mediates the differences in AhR responses among species [19,20] (Figure 1). AhR forms a complex with multiple dimers in the cytoplasm during the inactive state, including the HSP90 dimer, XAP2, and P23. After activation, the AhR complex relocates to the nucleus, combines with intranuclear ARNT, and forms the heterodimer AhR/ARNT [21]. At this point, AhR detaches from the protein complex and then binds to the DRE, recruiting coactivators to form an active transcription complex, which ultimately regulates the expression of some metabolic enzyme genes and other genes [22,23]. However, another pathway has also been observed, including AhR binding to other nuclear proteins, such as *KLF6* and *RelB,* to activate the expression of different downstream genes that regulate many processes, such as cell cycle regulation and growth differentiation [24,25], and can also be employed as coactivators of numerous nuclear transcription factors, such as E2F1 and ER [26,27]. Moreover, AhR can act as an atypical E3 ubiquitin ligase to regulate the degradation of steroid receptors such as estrogen receptor alpha (ERα), ERβ, and the androgen receptor [28] (Figure 2).

## 3. Species Differences of AHR Agonistic Effects

AhR agonists cause physiological and pathological manifestations, including guarding the intestinal barrier integrity, modulating immunity, cardiovascular function, liver damage, and thymic involution [29,30]. Moreover, potent AhR agonists can cause death at a single high dose [31]. From a genetic perspective, as a regulator of the cellular response to xenobiotics, the activation of AhR causes a change in the expression of many genes [32]. The following discussion on the species-specific responses of active AhR can be divided into the following two parts.

### 3.1. Species-Specific Morphological Responses to Active AhR

The morphological responses of AhR to endogenous and exogenous ligands are different, and species-specific responses have been observed in many ligands. Indigo and indirubin, the main components of Indigo naturalis, are AhR agonists and are used clinically to treat ulcerative enteritis. Extended exposure to these components in humans leads to blood in the stool and pulmonary artery hypertension, while these effects are not observed in mice [33,34,35]. Glucobrassicin is found naturally in vegetables such as broccoli, Brussel sprouts, and cabbage, which are metabolized by stomach acid to produce many high-affinity AhR ligands, such as indole-3-carbinol (I3C) [36]. Human exposure to I3C did not elicit demonstrable toxicity, but the perinatal exposure of pregnant rats to I3C induced reproductive abnormalities in male rat offspring [37]. Among all the known AhR ligands, 2,3,7,8-tetrachlorodibenzo-p-dioxin (TCDD) has the highest toxicity and potency. Acute TCDD toxicity in adult animals primarily occurs in the thymus, liver, nervous system, skin, and development [38]. However, most of these specific pathological phenomena are dependent on which species is studied, except for thymic atrophy, which is constantly observed among the studied mammals [39,40]. For example, the primary toxicity in mice is hepatic steatosis, while it is hepatocyte hypertrophy in rats [41]. The teratogenic effects of TCDD exposure were observed in hamsters and rats but not in guinea pigs [42]. TCDD also induced differential responses in choline metabolism, ALT, cholesterol, and triglycerides [43]. Among all vertebrates, fish are one of the most sensitive to TCDD exposure [44]. However, there are still significant variations in sensitivity within the same species. For example, in a comparison of the lethal potency of TCDD between the most sensitive fish species, bull trout (*Salvelinus confluentus*), and least sensitive, zebrafish (*Danio rerio*), we found that there was a 120-fold difference [45]. We compared the toxicity of TCDD to seven freshwater fish species during early life-stage development. All young fish were found to have edema and head and spinal deformities, but skin discoloration was observed only in some fathead minnows (*Pimephales promelas*) and medaka (*Oryzias latipes*) [46]. TCDD sensitivity studies to date have shown that all frogs and toads (*order Anura*), unlike mammals, fish, and birds, are incredibly insensitive to TCDD [47]. Several studies have suggested that American toads (*Bufo americanus*), leopard frogs (*Rana pipiens*), and embryos and tadpoles of green frogs (*Rana clamitans*) are 100- to 1000-fold less sensitive to TCDD-induced lethality than most fish species [48]. On the other hand, TCDD exposure in fish and birds highlights the cardiovascular toxicity of TCDD, which is manifested as inhibited definitive erythropoiesis, reduced cardiac function, and cardiac malformations [49]. A median lethal dose (LD_50_) of a single oral dose of TCDD also showed significant species differences. For example, the LD_50_ of guinea pigs for TCDD was 0.6–2.1 μg/kg [50], while that of hamsters was greater than 5000 μg/kg [51]. The LD_50_ also showed differences in the same animal branches. For example, the LD_50_ of Han/Wistar (*Kuopio*) rats was over 10,000 μg/kg compared to 17.7 μg/kg in Long-Evans (*Turku/AB*) rats [52]. The same observation was recorded in mice: C57BL/6 were 182 μg/kg, while it was 2570 μg/kg in DBA/2 [53]. Interestingly, human AhR is less responsive to TCDD than rodent AhR, while it retains a higher sensitivity toward indirubin, kynurenic acid, and other endogenous AhR activators [54,55,56].

### 3.2. AhR-Mediated Species-Specific Gene Expression Networks

The activation of AhR results in the induction of a gene battery. Different expression levels of these genes have been observed in vitro and in vivo in different species.

For the TCDD induction of CYP1A2 and CYP1A1 mRNA, human hepatocytes were 30- and five-fold less sensitive than rats, respectively [57]. Atypical AhR agonists, such as omeprazole, can induce the expression of *CYP1A1/2* through the transcriptional activation of AhR in human primary hepatocytes, while they have no such effect on rat primary hepatocytes [58]. To compare the differences in human and rodent responses to AhR, Edward Dere [59] treated human HepG2, mouse Hepa1c1c7, and rat H4IIE hepatoma cells with 10-nM TCDD for 24 h, followed by toxic genomics and a DNA microarray analysis. The results showed that, in HepG2 and Hepa1c1c7 cells, only 22 orthologous genes (9.3% in all HepG2 differentially expressed orthologous genes and 15.8% in Hepa1c1c7) were regulated by TCDD. A response in only eight differentially expressed orthologous genes in both Hepa1c1c7 (1.4%) and H4IIE (8.3%) cells was identified in the rodent platforms. GO terms related to lipid metabolism were enriched in the HepG2 and H4IIE cell lines, which was consistent with the results of liver gene enrichment in rats treated with TCDD in vivo [60]. Agnes L. Forgacs [61] compared TCDD treatment on the genome enrichment of human, mouse, and rat primary hepatocytes. Comprehensive time course (10-nM TCDD for 1, 2, 4, 8, 12, 24, and 48 h) studies identified mouse-specific enrichment that included lipid generation and metabolism genes, while the rat-specific functions were associated with intracellular lipid transport and localization. Michael B. Black [62] exposed human and rat primary hepatocytes to different concentrations of TCDD (0.00001 to 100 nM) for 24 h. The species-specific enrichment results showed that pyruvate and testosterone, as well as retinol biosynthesis and the metabolism pathways, were enriched only in human hepatocytes, but the glycolysis and gluconeogenesis metabolism pathways were enriched in rats, which was different from the results of Agnes L. Forgacs. Perhaps this difference was most likely related to the different sources of rat primary cells. Michael B. Black used primary hepatocytes derived from female rats, while Agnes L. Forgacs used male rats.

In vivo, gene expression profiles have a significant diversity of intraspecies and interspecies responses to AhR. A genomic data analysis showed that less than 20% of AhR-responsive orthologs were conserved in the livers of both Sprague–Dawley rats and C57BL/6 mice upon TCDD treatment [41]. Among all of the differentially expressed orthologs, the mouse gene function assembled on the lipid metabolism, while the rat gene function was associated with choline metabolism [43]. These results suggested the cause of mouse-specific liver lipid accumulation and rat-specific choline metabolism disorder after TCDD treatment [63]. During TCDD treatment in C57BL/6J mice and hAhR transgenic primary mouse hepatocytes, only 18% of the genes were significantly activated by mice and human AhR [18]. However, in the same species, different strains had specific response genes to AhR. Research on AhR-regulating transcriptomic changes in rats that were sensitive or resistant to TCDD revealed that animal-specific dioxin lethality could be related to a broad diversity of entire pathways, not only single genes [64].

## 4. Mechanisms for the Species Difference after AhR Activation

All ligands tend to interact with AhR in the LBD domain regardless of the species. In most species, the mechanism of AhR action is believed to be the same. Moreover, the associated proteins, such as HSP90 and ARNT, are remarkably similar, which motivates the question as to why different species have wildly different AhR responses. The ligands that enter the cytoplasm and bind to AhR then shuttle into the nucleus, identify the DRE sequence, recruit coactivators, and start downstream gene transcription. However, the binding affinity of the ligands to AhR, the recruited coactivator, and the DRE in the target gene all affect the final AhR response. Hence, we will introduce the mechanism of AhR species diversity from these aspects.

### 4.1. The Influence of the AhR-LBD Primary Structure on Species Diversity

The crucial inter- and intraspecies variations reported in the AhR response appear to be primarily due to differences in the primary structure of the AhR-LBD. The following will introduce the structural differences of the AhR-LBD from mammals, birds, fish, and frogs that have been more researched.

**Figure 3:**
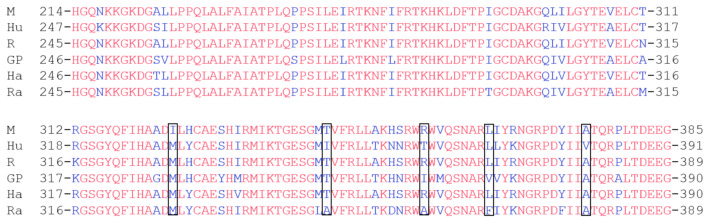
Interspecies multiple sequence alignment of the AhR-LBD. M, mouse; Hu, human; R, rat; GP, guinea pig; Ha, hamster; Ra, rabbit. Some AhR-LBD residues have been characterized by mutagenesis studies and could be responsible for the species-specific AhR response, highlighted in black.

Across species, the primary structure of the AhR-LBD is highly conserved. Multiple sequence alignment of the AhR-LBD domain from mice, humans, rats, guinea pigs, hamsters, and rabbits was performed (Figure 3). The sequence alignment showed that there are only a few amino acid differences, which is one of the reasons for the different levels of AhR activation. Among these different amino acid residues, Ala375 (in C57BL/6 mice) has been studied the most [65,66]. The site-directed mutagenesis of Ala375 to Val showed that the binding activity of AhR and TCDD was reduced [67]. In the AhR-LBD of DBA mice, this site corresponded to Val, which extensively decreased TCDD toxicity compared to C57BL/6 mice [68]. Interestingly, this phenomenon has also been found in humans. Compared with Neanderthals and Primates (expressing the AhR ancestral variant Ala381, which corresponds to Ala375 in mice), the AhR-derived variant Val381 in modern humans leads to a reduced AhR affinity to aryl hydrocarbons [69]. This observation could be due to the change in Ala375Val, which introduces a steric hindrance at the end of the AhR-LBD-modeled cavity, which, in turn, reduces the practical internal cavity volume and potentially affects ligand interactions with other fingerprint residues [70]. Omeprazole is an AhR agonist, and AhR activity following the omeprazole effect has shown differences among species. Compared to the AhR-LBD of omeprazole-sensitive rabbits and omeprazole-insensitive mice, we found that T353, F367, and M328 in rabbit AhR were responsible for omeprazole-mediated species-specific transactivation [71]. The pure antagonist 3-methoxy-4-nitroflavone of TCDD-induced AhR DRE binding in mouse hepatoma cells is a partial agonist in adenocarcinoma cells of guinea pigs [72]. What could contribute to the variations between these two mammalian species (mouse R355 in AhR and guinea pig I360 in AhR) is the amino acid residues in AhR-LBD [73]. More recently, indirubin, kynurenic acid, and other natural and presumed endogenous ligands have been shown to be more potent when binding to human AhR than rodent AhR [54,74,75]. However, the amino acid variations responsible for this observation are still elusive.

Birds have at least two AhR isoforms of which AhR1 exhibits a more dominant function than AhR2. Although the AhR1 of birds is highly conserved (>90%), the sensitivity of birds to TCDD shows high interspecies variations [76] (up to 1000-fold). For example, common terns (*Sterna hirundo*) and chickens (*Gallus gallus*) AhR share an incredibly high sequence identity (98%) in the amino acids of AhR-LBD. The only differences are in three amino acid residues (in chickens: Ala257, Ile324, and Ser380 compared to Thr258, Val325, and Ala381 in common terns). However, their sensitivity to TCDD is 80–250-fold different. A site-directed mutagenesis analysis found that only the two amino acids at positions 324 and 380 in chickens led to this difference in sensitivity [8]. Another study determined and compared amino acid sequences of the AhR1 LBD from 86 avian species; the results revealed that only amino acids at sites 324 and 380 affect the sensitivity of AhR1 to TCDD [77], which demonstrates that the volume of the AhR1-LBD cavity and the hydrogen bonds of the residues could be the reasons for the variations in sensitivity. For example, the reason for the high sensitivity of chickens could be that the hydrogen bond of the hydroxyl group of serine (CH3OH) at position 380 can interact with the hydrogen bond of two oxygen bridges of TCDD to stabilize the ligand receptor. However, when chicken AhR1 undergoes Ile324Val and Ser380Ala mutagenesis, the cavity volume of its AhRl-LBD increases, which reduces its sensitivity to TCDD [78].

Fish AhRs are classified into at least three distinct clades (AhR1, AhR2, and AhR3), in which each clade can have multiple isoforms. AhR2 has been considered to be the active form in fish research to date [79]. Jon A. Doering [80] found that white sturgeons (*Acipenser transmontanus*) were 3–30-fold more sensitive than lake sturgeons (*Acipenser fulvescens*) after exposure to different dioxin-like compounds based on the activation of AhR2, and these differences might result from differences in crucial amino acids at position 388 in the LBD of AhR2. White sturgeons (Ala-388) were compared to Thr-388 in lake sturgeons. Overall, the conservation in the AhR sequence of fish was less than 70% based on publicly available sequences. Moreover, the different AhR clades and isoforms in fish make it more complicated to predict the crucial AhR-LBD amino acid sequence that contributes to TCDD sensitivities among species. The effect of the frog AhR-LBD structure on TCDD sensitivity is not very clear.

Only some clues have been found thus far. Some frogs express two distinct AhR1 genes: AhR1α and AhR1β, the two AhR paralogs. Both AhR1α and AhR1β of salamanders and clawed frogs (*Xenopus laevis*) share 86% amino acid identity and exhibit extremely low TCDD affinity [81]. Some researchers found that residues N325, A354, and A370 within the LBD of *X. laevis* AHR1β are associated with a low TCDD affinity. When A354 was changed to serine, the EC_50_ for TCDD decreased more than 15-fold. When N325 was changed to serine, the EC_50_ declined three-fold [82].

### 4.2. The Influence of the AhR TAD Structure on the Species Diversity

In addition to the differences in amino acids at critical sites of the AhR-LBD, the lack of interspecies conservation of AhR TADs might result in species differences in the AhR activation effect. The subdomains (P/S/T, Q-rich, and acidic) of TAD have distinct and independent functions and a wide range of activation levels. However, the Q-rich subdomain appears to play a more significant role in species differences of AhR activation. The N-terminus of AhR is highly similar between guinea pigs that are sensitive to TCDD and hamsters that are resistant to TCDD. However, the TAD Q-rich region in guinea pigs is only half that of hamsters, which could be responsible for this difference [83]. In Han/Wistar (TCDD-resistant), the functionally essential Q-rich region is also substantially expanded [84]. Interestingly, it appears that a critical mutation in the intron sequence causes an alteration in the P/S/T structure in the AhR of the Han/Wistar, which brings about TCDD resistance [85]. Additionally, a distinct correlation between the LD_50_ values for TCDD and the number of glutamine residues in the Q-rich subdomain has been observed in mammalian species [86]. The molecular details of how the AhR TAD structure leads to such selective AhR responses are still unclear. Some authors thought that AhR differentially recognizes the LXXLL motif coactivators [20]. The LXXLL motif is a conserved signature sequence of the coactivator’s interacting domain. It enables the interactions of ligand-dependent nuclear receptors. The LXXLL motifs of some coactivators, such as nuclear receptor-interacting protein-1 (RIP140) and steroid receptor coactivator 1 (SRC-1), interact with the Q-rich subdomain of the AhR TAD, and different AhR TAD structures can differentially interact with coactivators [87].

### 4.3. The Influence of DRE Cores on Species Differences

Once the AhR complex enters the nucleus, it binds to the DRE 5′-TNGCGTG-3′ gene promoter, promoting downstream gene transcription. The efficacy of the transcription of this sequence increases when the “N” is cytosine or thymine (C or T) in the sequence rather than when it is guanine or adenine (G or A) [88]. Manabu Nukaya [89] used homologous recombination to produce model mice in which CYP1A1 and CYP1A2 lacked the DRE fragment. The results showed that, after being given dioxin, the hepatic CYP1A1 mRNA levels in the WT mice were increased the most (35-fold), and in the DRE^+/−^ mice, the increase was 18-fold, while the induction of CYP1A1 essentially eliminated the increase in DRE^−/−^ mice. Furthermore, Ken-Ichi T. Suzuki [90] emphasized that resistance to TCDD in amphibians can be explained by differences in the number of DREs and their localization in CYP1A1 gene promoters. This finding demonstrated that DREs play an essential role in the AhR response. The genome-wide analysis results showed substantial differences in the distribution, location, and number of DREs in the same AhR target gene among humans, mice, and rats [91,92]. To explore the relationship between these factors and species differences, Edward Dere [59] induced AhR of a hepatoma cell line in a mouse (Hepa1c1c7), human (HepG2), and rat (H4IIE) with TCDD. A microarray analysis showed different DRE cores within the same genes (*GSTA5* and *CCND1ID3*) of each species. The number of DRE core sequences of these genes was proportional to the degree of TCDD response. Other researchers found that increasing the number of proximal promoter DREs caused an elevation in the expression levels of the direct target genes [93]. Interestingly, not all DREs are able to initiate gene expression. Gary Zeruth studied the function of putative DREs in *CYP1A* genes in mammals and fish and showed that induction also depends on the occurrence of binding sites for other transcription factors and coactivators in the vicinity of the DREs [94].

To summarize, although the DRE core sequences of genes are highly conserved among species, the differences in the DRE number, adjacent sequences, and specific bases in the DRE core lead to different activation effects among species.

## 5. Conclusions

AhR has a long evolutionary history of enabling cells to adapt to various conditions. This protein can interact with compounds from the environment, diet, and microbiome and is ubiquitous in many human and animal tissues, which signifies that AhR has a vital role in growth and development. Early studies on AhR mainly focused on the toxicological reactions caused by dioxins and other environmental toxicants with a high affinity for AhR without paying attention to the possible species differences in AhR toxicity. Later, toxicologists found species differences in AhR toxicity, and this phenomenon has also been observed regarding recently discovered endogenous AhR ligands. With the discovery of the new role of AhR in mammals, many AhR ligands with well-defined health-promoting effects have been researched as potential drugs to target AhR for the treatment of many diseases. Given the known physiological differences between species after AhR activation, much of the data generated from inbred rodent model systems cannot be directly extrapolated in human cases. Therefore, it is necessary to elucidate the biochemical and molecular mechanisms of exogenous and endogenous ligands that can differentially regulate AhR functionality and its downstream responses. We reviewed the species-specific responses of AhR based on gene expression, morphological responses, and LD_50_ between species, and then, we inserted some of the most accepted explanation attempts, including species-specific AhR-LBD, AhR TAD, and DRE cores, to explain the mechanisms of these differences. In addition, amino acid deletions outside of the LBD and differences in the expression of AhR, AhRR, and coactivators in different species also affect the species-specific responses of AhR. However, at present, research on the species differences of activated AhR and its mechanisms is only the tip of the iceberg, and finding more specific and comprehensive mechanisms is still required.
